# Host Genetic Variants Linked to COVID-19 Neurological Complications and Susceptibility in Young Adults—A Preliminary Analysis

**DOI:** 10.3390/jpm13010123

**Published:** 2023-01-06

**Authors:** Anastasiya Kazantseva, Renata Enikeeva, Zalina Takhirova, Yuliya Davydova, Rustam Mustafin, Sergey Malykh, Alexandra Karunas, Alexander Kanapin, Elza Khusnutdinova

**Affiliations:** 1 Institute of Biochemistry and Genetics—Subdivision of the Ufa Federal Research Centre of the Russian Academy of Sciences, 450054 Ufa, Russia; 2 Laboratory of Neurocognitive Genomics, Department of Genetics and Fundamental Medicine, Ufa University of Science and Technology, 450076 Ufa, Russia; 3 Department of Medical Genetics and Fundamental Medicine, Bashkir State Medical University, 450008 Ufa, Russia; 4 Psychological Institute, Russian Academy of Education, 125009 Moscow, Russia; 5 Department of Psychology, Lomonosov Moscow State University, 125009 Moscow, Russia; 6 Centre for Computational Biology, Peter the Great St. Petersburg Polytechnic University, 195251 St. Petersburg, Russia

**Keywords:** coronavirus, COVID-19, genome-wide association study (GWAS), neurological symptoms, mental health, gene

## Abstract

To date, multiple efforts have been made to use genome-wide association studies (GWAS) to untangle the genetic basis for SARS-CoV-2 infection susceptibility and severe COVID-19. However, data on the genetic-related effects of SARS-CoV-2 infection on the presence of accompanying and long-term post-COVID-19 neurological symptoms in younger individuals remain absent. We aimed to examine the possible association between SNPs found in a GWAS of COVID-19 outcomes and three phenotypes: SARS-CoV-2 infection, neurological complications during disease progression, and long-term neurological complications in young adults with a mild-to-moderate disease course. University students (N = 336, age 18–25 years, European ancestry) with or without COVID-19 and neurological symptoms in anamnesis comprised the study sample. Logistic regression was performed with COVID-19-related phenotypes as outcomes, and the top 25 SNPs from GWAS meta-analyses and an MR study linking COVID-19 and cognitive deficits were found. We replicated previously reported associations of the *FURIN* and *SLC6A20* gene variants (OR = 2.36, 95% CI 1.31–4.24) and OR = 1.94, 95% CI 1.08–3.49, respectively) and remaining neurological complications (OR = 2.12, 95% CI 1.10–4.35 for *SLC6A20*), while *NR1H2* (OR = 2.99, 95% CI 1.39–6.69) and *TMPRSS2* (OR = 2.03, 95% CI 1.19–3.50) SNPs were associated with neurological symptoms accompanying COVID-19. Our findings indicate that genetic variants related to a severe COVID-19 course in adults may contribute to the occurrence of neurological repercussions in individuals at a young age.

## 1. Introduction

Severe acute respiratory syndrome coronavirus 2 (SARS-CoV-2), which causes coronavirus disease 2019 (COVID-19), has provoked a tremendous burden on society worldwide, including considerable economic costs and a high mortality and morbidity rate. In addition, the long-term consequences of the disease and its effect on various systems of the body can negatively affect many aspects of an individual’s life.

The effect of the SARS-CoV-2 virus on the respiratory system, cardiovascular system, and gastrointestinal tract has been repeatedly demonstrated [1,2]. Together with epidemiological data on disease severity related to anthropometric parameters and concomitant chronic pathology [1], the role of genetic factors in COVID-19 illness and disease severity has been reported (for review, see [2,3,4]). Twin studies have elucidated a substantial genetic influence for predicted COVID-19, with a concordance rate of 80% in MZ twins [4]. The largest study was conducted by the COVID-19 Host Genetics Initiative (COVID19 HGI) (released on 15 June 2021) [5], which established a link between host genes and three COVID-19 outcomes in a genome-wide association study (GWAS) meta-analysis. A list of genetic variants associated with overall disease susceptibility (112,612 cases, 2,474,079 controls, mainly Europeans), hospitalization due to COVID-19 (24,274 cases, 2,061,529 controls, mainly Europeans), and critical COVID-19 (8779 cases, 1,001,875 controls, mainly Europeans) was reported [5]. In summary, the following genetic loci were shown to have genome-wide statistical significance in several COVID-19 GWAS: the blood type *ABO* locus; a cluster of immune system genes (*SLC6A20*, *SMRR1,* and the immunoglobulin lambda locus) [6,7]; a gene cluster encoding antiviral restriction enzyme activators neighboring the *TYK2*, *DPP9*, and *IFNAR2* genes [8]; the *IVNS1ABP*/*SWT1* gene cluster [7]; and the *MYH14*, *SETX*, *ATXN1*, and *SCN11A* genes [9]. A significant association of exonic variants in the *ACE2*, *TMPRSS2*, and *FURIN* genes with COVID-19 incidence was detected by another research group using whole-exome sequencing [10].

Together with the well-established negative effects of SARS-CoV-2 on the pulmonary system, gastrointestinal tract, and cardiovascular system, the virus can also penetrate the blood–brain barrier, affect both neurons and glial cells, and cause neurological complications [3]. Different pathomechanisms underlie the development of neurological impairments, including post-COVID-19 syndrome, such as chronic fatigue, cognitive dysfunction, mood and sleep disorders, pain syndromes, and smell and taste impairments [2]. Previous research has indicated a causal relationship between genetically mediated hospitalized and critical COVID-19 and later memory problems, such as Alzheimer’s disease (AD) and other types of dementia [11]. Interestingly, the *ApoE* ε4 allele, which was linked to cognitive impairment and functioning [12], was also reported as being associated with an increased risk of severe COVID-19 [13] and infection incidence [14].

An exaggerated susceptibility rate for SARS-CoV-2 infection, more severe forms of the disease [15], and the hyperactivity of immune responses [16] have been detected in adults and the elderly. However, pronounced neurological problems accompany the disease course and manifest as long-term repercussions even in young adults and children [17,18]. Nonetheless, data on the genetic-related effects of SARS-CoV-2 infection on the presence of accompanying neurological symptoms and long-term neurological consequences in younger individuals are lacking. Although several specific findings of gene variants implicated in disease susceptibility [19] and severe COVID-19 [20] in children have been reported, it was also suggested that coronaviral infection may result in an enhanced rate of mental health problems later in life [2]. Therefore, the identification of plausible host genetic variants associated with an elevated risk of developing neurological repercussions during SARS-CoV-2 infection and over a long-term period in younger individuals is of relevance. It will help to develop practical recommendations that can be given to genetically predisposed individuals to reduce the probable mental problems caused by SARS-CoV-2 infection.

As previously reported, neurological impairments are more frequently associated with a severe COVID-19 course, including critical illness and hospitalization [2,21,22]. Therefore, we hypothesized that young adults with long-term neurological symptoms could be the carriers of risky SNP alleles, which have previously been associated with mental problems and severe disease courses in adults.

Herein, we aimed to examine a possible association between SNPs identified in a GWAS of COVID-19 outcomes and three phenotypes, i.e., SARS-CoV-2 infection, neurological complications during disease progression, and in long-term neurological complications, in young adults with a mild-to-moderate disease course.

## 2. Materials and Methods

### 2.1. Participants

A sample of university students (N = 336; mean age ± SD: 21.3 ± 1.2 years; age range: 18–25 years; 77% women) from the Volga–Ural region of Russia was collected during the first three waves of the COVID-19 pandemic. All participants were of European ancestry (22% Russians, 61% Tatars, and 17% of mixed ethnicity). The exclusion criteria were a self-reported individual or family history of any psychiatric disorder. The following data were obtained from the participants: sex, ethnic background, SARS-CoV-2 infection in anamnesis, dates of coronaviral infection, clinical symptoms accompanying the disease course, and neurological complications that appeared within 6 months and were maintained over a prolonged period after COVID-19 recovery (up to one year). Disease susceptibility (cases) was assigned based on a positive PCR test or a reported suspected COVID-19 illness. All cases indicated a mild (absence of any shortness of breath, dyspnea, or abnormal chest imaging) or moderate (individuals with a febrile temperature lasting for at least one week or lower respiratory tract inflammation but SpO_2_ > 94%) disease course without hospitalization, as previously classified [23]. Subjects were classified as resistant to SARS-CoV-2 infection (controls) if they reported an absence of COVID-19 infection during the first three waves of the COVID-19 pandemic prior to their individual vaccination against SARS-CoV-2. Information regarding the clinical symptoms accompanying coronaviral disease was obtained for disease cases preceding individual vaccination. Neurological symptoms included severe headache, memory problems, anosmia/dysosmia, ageusia/dysgeusia, insomnia and sleep problems, anxiety, and depression manifested during the course of the disease, or later after recovery, and maintained for a period of at least one year. 

The study was approved by the Biological Ethics Committee at the Institute of Biochemistry and Genetics—Subdivision of the Ufa Federal Research Centre of the Russian Academy of Sciences (Ufa, Russia) (protocol code 19, date of approval, 25 November 2021). Written informed consent was obtained from all participants after they were acquainted with the procedures. All participants were informed about the voluntary and confidential nature of their participation. All procedures performed were in accordance with the ethical standards of the institutional and/or national research committee and with the 1964 Helsinki Declaration and its later amendments or comparable ethical standards.

### 2.2. SNPs Selection and Genotyping

Genomic DNA was extracted from the leukocytes of enrolled volunteers using a standard phenol–chloroform technique. We selected the top SNPs reported in two major meta-analyses from the COVID-19 HGI describing the genetic impact on COVID-19 susceptibility, hospitalization, and critical illness [5,24] and one Mendelian randomization study linking COVID-19 and cognitive impairment [11]. We selected significant genetic variants from the MR study, including *NR1H2* rs1405655, *MUC5B* rs35705950, *SLC6A20* rs2531743, *KEAP1* rs45524632, and *ABO* rs635634. A final set of 25 SNPs from these studies was formed based on their presence in the Infinium Global Screening Array-24 v.3.0 Kit (Illumina, San Diego, California, USA), which was initially used for genotyping on the Illumina IScan platform (Genotek, Russia). For several SNPs identified by HGI, that were absent in the genotyping array, we obtained data on the genotypes of the closest proxy SNPs (https://ldlink.nci.nih.gov, accessed on 20 October 2022, r^2^ > 0.7) present in the 1000 Genomes Project (www.ldlink.nci.nih.gov (accessed on 20 October 2022)). A complete list of SNPs examined within the framework of this study and their relevance to the genes is presented in the Supplementary Information (Table S1). The chosen SNPs resided in the *SMRR1*, *IVNS1ABP*, *SLC6A20*, *LZTFL1*, *HLA-G*, *CCHCR1*, *NOTCH4*, *ABO*, *MUC5B*, *OAS1*, *OAS3*, *FURIN*, *DPP9*, *TYK2*, *KEAP1*, *APOE*, *NR1H2*, and *TMPRSS2* genes. Some of the SNPs overlapped among the studies [5,11,24].

### 2.3. Statistical Analysis

To test for differences in the distribution of COVID19-related outcomes between the sexes, we used a chi-square test. A correlation analysis was conducted between the phenotypes using Spearman’s rank correlation coefficient. 

A series of logistic regression analyses were performed to address the association of COVID-19 GWAS SNPs with the three examined disease-related outcomes in mild-to-moderate disease cases. In the regression models, reported previous infection, neurological symptoms accompanying infection, and reported neurological complaints that appeared or were maintained within one year of SARS-CoV-2 infection were included as independent outcomes. In the statistical models, the genotyping data on the investigated SNPs served as the independent variables. In addition, we examined both log-additive and dominant logistic regression models, which evaluate the allele effect in a dose-dependent (AA vs. aA vs. aa) and a dominant manner (AA + aA vs. aa), respectively. For the SNPs with a significant effect on COVID-19 outcomes, we calculated ORs within 95% confidence intervals. To estimate the effect of multiple gene variants on COVID-19 outcomes, we designed logistic regression models with the inclusion of all examined SNPs, followed by the selection of the best prediction model based on the backward elimination procedure, which was chosen on the basis of the best Akaike information criterion (AIC) and *p*-value in R [25]. All statistical analyses were carried out with R v.4.1.2 and PLINK v.1.9.

## 3. Results

### 3.1. Statistical Analysis between Clinical and Demographic Data

The clinical characteristics of the examined sample were as follows: history of COVID-19 infection (67%), accompanying neurological symptoms (70% of cases) and anosmia (65% of cases) within the disease course, and long-term neurological consequences (36% of cases). In the first stage of the analysis, we examined whether there were sex-specific differences in three COVID-19-related outcomes: COVID-19 susceptibility, the presence of neurological complications accompanying the disease course, and those maintained for a prolonged period (within at least one year) after recovery. No statistically significant differences in either disease susceptibility or accompanying and future neurological symptoms (including anosmia) were identified between the sexes. However, there was a trend toward a difference in infection susceptibility between men and women (χ^2^ = 3.27, *p* = 0.07), with a higher infection prevalence in men compared to women.

From the correlation analysis, we observed a significant positive correlation between the presence of neurological symptoms during COVID-19 illness and those that appeared/were maintained within a prolonged period after recovery (r = 0.44, *p* < 0.01), as well as between accompanying anosmia and later neurological consequences (r = 0.45, *p* < 0.01).

### 3.2. Association Analysis between SNPs and COVID-19 Outcomes

As a primary step, we checked all analyzed SNPs for their congruence with the Hardy–Weinberg equilibrium (HWE). SNPs that failed the HWE check were excluded from further association analysis (rs61735789, rs117696554, and rs2838046 in the *TMPRSS2* gene, rs657152 and rs635634 in the *ABO* gene).

**Figure 1:**
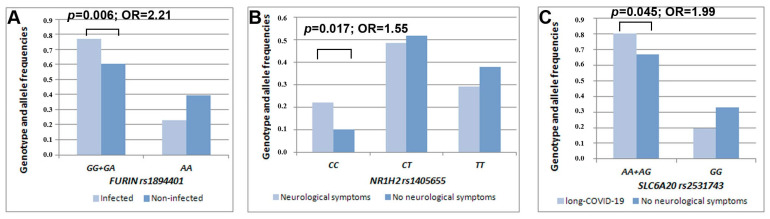
Distribution of the genotype/allele frequencies of *FURIN* rs1894401 depending on COVID-19 infection susceptibility (**A**), *NR1H2* rs1405655 depending on the presence/absence of accompanying neurological symptoms (**B**), and *SLC6A20* rs2531743 depending on the presence/absence of neurological consequences over a prolonged period (**C**) in the examined sample of mild-to-moderate COVID-19 cases.

Our statistical analysis detected an association between rs1894401 in the *FURIN* gene and COVID-19 infection (β = 2.74, *p* = 0.006). Individuals carrying the rs1894401 G-allele (GG + GA genotype) had a higher risk of infection than those carrying the AA genotype (OR = 2.21, 95% CI 1.25–3.88) (Figure 1). Another statistically significant result was obtained for rs1405655 residing in the *NR1H2* gene, where the rs1405655 C-allele was related to an increased risk of manifesting neurological symptoms during COVID-19 illness in a dose-dependent manner (β = 2.37, *p* = 0.017, OR = 1.55, 95% CI 1.08–2.23). Furthermore, rs2531743 in the *SLC6A20* gene was associated with long-term neurological complaints after recovery from COVID-19 illness (β = 1.99, *p* = 0.045). To be more precise, carriers of the rs2531743 A-allele had a higher risk of manifesting later neurological symptoms than those bearing the GG genotype (OR = 1.99, 95% CI 1.01–3.91) (Table 1, Figure 1).

We also observed a trend for the association between the rs4290734 G-allele residing in the *TMPRSS2* gene (β = 1.76, *p* = 0.078) and the presence of neurological symptoms in our cohort of mild COVID-19 cases (Table 2).

In order to prioritize genetic variants by their significance with respect to COVID-19 susceptibility and neurological symptoms, we examined the combined effect of various SNPs via a series of logistic regressions (under a general linear model procedure). Initially, all SNPs and sexes were included as potential covariates. As a result of backward elimination, the final model predicting enhanced SARS-CoV-2 infection susceptibility included the *FURIN* rs1894401 AA genotype compared to the GG + GA genotypes (β = 0.85, *p* = 0.0038, OR = 2.36, 95% CI 1.31–4.24) and *SLC6A20* rs2531743 GG-genotype compared to AA+AG genotypes (β = 0.66, *p* = 0.026, OR = 1.94, 95% CI 1.08–3.49) (Table 3). The best model for predicting the occurrence of neurological symptoms accompanying the COVID-19 disease course consisted of the *NR1H2* rs1405655 TT genotype compared to the CC genotype (β = 1.09, *p* = 0.0059, OR = 2.99, 95% CI 1.39–6.69) and the *TMPRSS2* rs4290734 AA genotype compared to the AG genotype (β = 0.71, *p* = 0.0099, OR = 2.03, 95% CI 1.19–3.50) (Table 3). The best model for predicting the development of neurological complications remaining within one year of disease consisted of the *FURIN* rs1894401 AA genotype compared to the GG + GA genotypes (β = 0.58, *p* = 0.085, OR = 1.79, 95% CI 0.93–3.61) and the *SLC6A20* rs2531743 GG genotype compared to the AA + AG genotypes (β = 0.75, *p* = 0.031, OR = 2.12, 95% CI 1.10–4.35) (Table 3). Although *FURIN* rs1894401 demonstrated a non-significant effect in the last model (*p* < 0.05), it had to be controlled for in the regression model based on the backward elimination procedure.

## 4. Discussion

Our analysis of the clinical manifestations of COVID-19 in mild and moderate cases in young adults points to the presence of positive correlations between neurological complications during the disease course and future neurological impairments, including severe headache, memory deficit, concentration problems, dysosmia, dysgeusia, insomnia/compromised sleep, anxiety, and depression. Even the presence of anosmia, which is a frequent clinical manifestation of COVID-19, was correlated with the mentioned set of neurological problems later in life in our sample. As previously suggested, such disturbances in the olfactory system could be related to long-term changes in the brain [26]. To date, an increased number of mental problems and neuropsychiatric complications (schizophrenia, affective disorders, Alzheimer’s disease, PTSD, etc.) attributed to COVID-19 illness over a prolonged period have been observed in long COVID-19 cases (for review, see [17]). However, long-term neurological sequelae resulting from SARS-CoV-2 infection have been shown to persist after one year, even in children and adolescents [18]. In this regard, the identification of biological markers (including genetic variants) associated with neurological complaints in younger individuals is of relevance.

In the present preliminary study analyzing 25 SNPs, we confirmed the impact of genetic variants in the *FURIN* and *SLC6A20* genes on reported SARS-CoV-2 infection susceptibility. Moreover, SNPs in the *NR1H2*, *TMPRSS2*, and *SLC6A20* genes, which were previously linked to COVID-19 hospitalization and cognitive decline, were related to neurological symptoms accompanying the COVID-19 disease course, as well as those remaining over a long-term period after recovery in our examined group of young adults.

We selected rs1894401 in the *FURIN* gene as a proxy to rs6226 (c.1851G > C), which was examined in a previous meta-analysis of COVID-19 susceptibility [24]. Initially, this SNP was detected via whole-exome sequencing in a Spanish population of patients with familial multiple sclerosis [10]. The observed implication of the *FURIN* gene in COVID-19 susceptibility is not surprising, since it encodes a proprotein convertase that activates various regulatory proteins, including the SARS-CoV-2 spike (S) protein. Furin, together with TMPRSS2 (transmembrane protease serine 2), is responsible for the cleavage of the SARS-CoV-2 S protein, which represents the SARS-CoV-2 co-receptor, regulates its entry into host cells [27], and is related to an enhanced risk of infection [28]. Several studies have examined the impact of *FURIN* SNPs on COVID-19-related phenotypes. It was demonstrated that alleles associated with higher protein expression (rs6224 T and rs4702 A) were the markers of COVID-19-implicated death [29]. Interestingly, the rs1894401 G-allele is correlated with the rs6224 risky T-allele (strongly linked, r^2^ = 0.941) and the rs4702 A-allele (r^2^ = 0.645). Therefore, we suggest that the rs1894401 G-allele, which was associated with an increased risk of SARS-CoV-2 susceptibility in the present study, is also likely linked to the overexpression of the *FURIN* gene.

A previous Mendelian randomization study demonstrated the presence of a positive genetic correlation between the dementia-like phenotype and COVID-19 hospitalization [11]. This observation is in line with our findings, which identified an association between rs1405655 in the *NR1H2* gene and the presence of neurological symptoms during the COVID-19 disease course. It should be noted that this SNP was selected as significant from a GWAS-based meta-analysis of three COVID-19-related phenotypes: critical illness, disease susceptibility, and hospitalization [5]. In the meta-analysis of data from 49,562 cases and two million controls, the rs1405655 C-allele was confirmed to be associated with disease severity at a genome-wide association level. Our findings also indicated that the rs1405655 C-allele was associated with an increased risk of developing neurological complications during COVID-19 progression, even in young adults with a mild-to-moderate disease course. In turn, the association observed in the present study is not surprising, as statistically significant genetic correlations have been indicated between COVID-19 hospitalization and several mental health issues, such as depression, insomnia, and ADHD [5]. At the molecular level, the *NR1H2* gene (also known as *LXRB*) encodes liver X receptor beta, which belongs to a subfamily of nuclear receptors regulating macrophage function, lipid homeostasis, and inflammation. It was shown that LXRβ has a functional implication in the development of cognitive impairments (such as Alzheimer’s disease) partly due to its key role in Aβ accumulation and cholesterol homeostasis [30]. Furthermore, the *NR1H2* gene is located in the same high-LOD score region for AD as the *ApoE* gene, whose role in the development of AD has been reported in numerous studies and was confirmed in a recent multiomics study [31]. Association studies examining the *NR1H2* gene in AD patients seem to be in line with our findings, as the rs1405655 C-allele was shown to segregate in families with this cognitive impairment [30]. As a result, our findings suggest that one of the molecular pathways associated with the manifestation of neurological symptoms during mild or moderate COVID-19 illness is related to impaired cholesterol homeostasis and the downregulation of *NR1H2* gene expression.

The *TMPRSS2* gene, which is responsible for SARS-CoV-2 penetration into the cell [3], has been repeatedly implicated in COVID-19-related outcomes [10,24,32]. Nevertheless, in the present study we observed a combined effect of *TMPRSS2* rs4290734 with *NR1H2* rs1405655 on accompanying neurological complications. A previous study identified a proxy SNP (*TMPRSS2* rs17854725 or c.879T > C) in a whole-exome scan of COVID-19 patients with familial multiple sclerosis [10], pointing to the possible involvement of the *TMPRSS2* gene in cognitive-related phenotypes.

The final finding of the present study was the association of rs2531743—located upstream of the *SLC6A20* gene (also known as *SIT1*)—with COVID-19 susceptibility (as a combined effect with *FURIN* rs1894401) and neurological complications, which appeared in six months after COVID-19 recovery and was maintained within at least one year. Initially, we selected this genetic variant as part of a set of SNPs in the MR study, which demonstrated a causal relationship between COVID-19 hospitalization and AD development [11]. A recent meta-analysis also demonstrated the role of the *SLC6A20* gene (based on rs11385942) in severe COVID-19 outcomes [24]. The *SLC6A20* gene encodes a protein that is involved in the transport of amino acids through cell membranes and is responsible for SARS-CoV-2 entry into the cell [32]. To be more precise, rs2531743 is located at 3p21.31, which has been previously linked to COVID-19 outcomes in eleven GWAS. SLC6A20 has been shown to interact with ACE2 in the membrane of small intestinal enterocytes, suggesting a relationship with COVID-19 illness [23]. Recent research has also demonstrated a link between SARS-CoV-2 sensitivity and *SLC6A20* gene variants in Vietnamese [23] and Native American [33] populations. In addition, it was suggested that SLC6A20 is a novel regulator of glycine levels [34]. This molecule is known for its impact on the central nervous system [35] and for having a beneficial effect on the proinflammatory cytokine secretion induced by SARS-CoV-2 infection [34].

The main strength of our preliminary study is the simultaneous analysis of a large set of COVID-19-related SNPs in a sample homogenous in age, level of education, and place of residence. However, the present study has several limitations. First, since the sample size was moderate, the observed associations are preliminary and have to be interpreted with caution. Second, since the major hypothesis of our study was to test for the relevance of SNPs with respect to neurological symptoms, we focused mainly on SNPs previously examined in the MR study linking COVID-19 illness to cognitive impairments. Third, we could not rule out whether the same direction of the SNPs’ effect would be present in individuals with a severe disease course and/or other age groups. Finally, due to the preliminary nature of our research, we did not perform any correction for multiple testing. Therefore, we cannot exclude the possibility of false-positive associations. Future directions in this regard include a replication study employing a larger sample size of the same population (young adults and children). In turn, it is of particular interest in future research to investigate the presence of neurological complications in the examined cohort under a longitudinal paradigm.

## 5. Conclusions

In summary, the present study, based on previous GWAS meta-analyses of COVID-19 susceptibility, hospitalization, and disease severity, partially replicated the implication of a genetic variant in the *FURIN* gene in SARS-CoV-2 susceptibility. Moreover, SNPs in the *NR1H2* and *SLC6A20* genes, which have been previously linked to COVID-19 hospitalization and AD, were related to neurological sequelae accompanying even a mild-to-moderate disease course, and those that appeared within six months and were maintained over a long-term period after recovery, in young adults.

Our findings indicate that alleles associated with the overexpression of the *FURIN* gene were responsible for higher susceptibility to SARS-CoV-2 infection in a general cohort of young adults. In addition, alleles linked to the downregulation of the *NR1H2* gene and impaired cholesterol homeostasis were found to contribute to the manifestation of neurological symptoms during mild-to-moderate COVID-19 illness. Finally, the indirect implication of the *SLC6A20* and *FYCO1* genes in the occurrence or maintenance of neuropsychiatric symptoms as long-term post-COVID-19 complications was bolstered by the reported regulation of the glycine-linked functioning of the central nervous system and proinflammatory cytokine secretion by SLC6A20.

## Figures and Tables

**Table 1:** Characteristics of the study sample.

Parameters	COVID-19 Patients N = 225	Neurological Symptoms ^1^ N = 158	Long COVID-19 ^2^ N = 81	Without Complications ^3^ N = 67	Controls N = 111
Sex, n (%)					
Women	173 (76.9)	119 (75.3)	64 (79.0)	46 (68.7)	93 (83.8)
Men	52 (23.1)	39 (24.7)	17 (21.0)	21 (31.3)	18 (16.2)
COVID-19 severity, n (%)					
Mild	185 (82.2)	126 (79.7)	81 (75.3)	60 (89.6)	-
Moderate	40 (17.8)	32 (20.3)	20 (24.7)	7 (10.4)	-
Neurological symptoms, n (%) severe headache	-	23 (14.6)	0	-	-
memory problems	-	3 (1.9)	63 (77.8)	-	-
anosmia/dysosmia	-	147 (93.0)	11 (13.6)	-	-
ageusia/dysgeusia	-	35 (22.1)	2 (2.5)	-	-
sleep problems	-	5 (3.2)	20 (24.7)	-	-
depression	-	5 (3.2)	7 (8.6)	-	-

^1^ Individuals with neurological complications accompanying the disease course; ^2^ individuals with neurological complaints that appeared and were maintained for a prolonged period (within at least one year) after COVID-19 recovery; ^3^ individuals without neurological complications during and after COVID-19 illness.

**Table 2:** Logistic regression analysis (log-additive models) demonstrating the effects of examined SNPs on COVID-19-related outcomes.

rsID	Gene Name	EA/NEA	SARS-CoV-2 Infection			Accompanying Neurological Symptoms			Long-Term Neurological Symptoms		
			EAF Cases/Controls	β	*p*-Value	EAF Cases/Controls	β	*p*-Value	EAF Cases/Controls	β	*p*-Value
rs114067890	*SMRR1*	G/A	0.01/0	<0.01	0.99	0.01/0	0.62	0.53	0.01/0.01	0.32	0.74
rs10911734	*IVNS1ABP*	T/C	0.30/0.37	−1.41	0.15	0.31/0.34	−0.65	0.51	0.30/0.33	−0.57	0.56
rs2531743	*SLC6A20*	A/G	0.50/0.44	1.17	0.24	0.49/0.47	0.47	0.63	0.55/0.46	1.99	**0.045** ^c^
rs17078348	*LZTFL1*	G/A	0.09/0.09	<0.01	0.99	0.10/0.09	0.40	0.68	0.11/0.09	0.55	0.57
rs9380142	*HLA-G*	G/A	0.23/0.29	−1.30	0.19	0.21/0.28	−1.70	0.09	0.26/0.25	0.25	0.80
rs112640945	*CCHCR1*	T/C	0.15/0.16	−0.04	0.96	0.15/0.16	−0.48	0.63	0.14/0.16	−0.65	0.51
rs3131294	*NOTCH4*	A/G	0.17/0.15	0.36	0.71	0.17/0.15	0.67	0.49	0.14/0.16	−0.52	0.59
rs657152	*ABO*	A/C	0.44/0.46	-	-	0.45/0.44	-	-	0.47/0.44	-	-
rs505922	*ABO*	C/T	0.39/0.42	−0.82	0.40	0.42/0.38	0.82	0.41	0.42/0.39	0.70	0.48
rs635634	*ABO*	T/C	0.39/0.42	-	-	0.38/0.42	-	-	0.35/0.41	-	-
rs35705950	*MUC5B*	T/G	0.13/0.16	−0.60	0.54	0.13/0.15	−0.41	0.67	0.16/0.14	0.63	0.52
rs4766664	*OAS1*	T/G	0.28/0.21	1.00	0.31	0.29/0.25	0.98	0.32	0.30/0.26	0.88	0.37
rs10735079	*OAS3*	G/A	0.31/0.32	−0.08	0.93	0.34/0.29	1.19	0.23	0.26/0.33	−1.41	0.14
rs1894401	*FURIN*	G/A	0.50/0.39	2.74	**0.006** ^a^	0.50/0.44	1.52	0.12	0.49/0.46	0.61	0.54
rs2109069	*DPP9*	A/G	0.21/0.20	0.25	0.79	0.21/0.20	0.18	0.85	0.23/0.20	0.66	0.50
rs2304256	*TYK2*	A/C	0.13/0.18	−1.53	0.12	0.14/0.15	−0.42	0.67	0.17/0.14	0.79	0.42
rs45524632	*KEAP1*	A/C	0.04/0.04	−0.08	0.93	0.02/0.05	−1.40	0.16	0.03/0.04	−0.41	0.67
rs769449	*APOE*	A/G	0.11/0.10	0.26	0.79	0.12/0.09	0.97	0.32	0.11/0.11	0.03	0.97
rs7412	*APOE*	T/C	0.20/0.22	−0.71	0.47	0.19/0.22	−0.74	0.45	0.18/0.21	−0.72	0.47
rs1405655	*NR1H2*	C/T	0.43/0.37	1.15	0.24	0.46/0.36	2.37	**0.017** ^b^	0.42/0.41	0.34	0.73
rs61735789	*TMPRSS2*	A/G	0.10/0.11	-	-	0.11/0.10	-	-	0.11/0.11	-	-
rs12329760	*TMPRSS2*	T/C	0.31/0.31	<0.01	0.99	0.30/0.31	−0.22	0.81	0.29/0.31	−0.52	0.60
rs4290734	*TMPRSS2*	G/A	0.31/0.26	1.22	0.22	0.33/0.26	1.76	0.078	0.32/0.29	0.64	0.51
rs117696554	*TMPRSS2*	A/G	0.19/0.18	-	-	0.18/0.17	-	-	0.15/0.18	-	-
rs2838046	*TMPRSS2*	G/A	0.31/0.31	-	-	0.33/0.29	-	-	0.30/0.31	-	-

Abbreviations: EA/NEA—effect (minor) allele/non-effect (major) allele; EAF—effect allele frequency; β—regression coefficient. Statistically significant *p*-values are highlighted in bold. a—values under the dominant model (OR = 2.21, 95% CI 1.25–3.88); b—values under the additive model (OR = 1.55, 95% CI 1.08–2.23); c—values under the dominant model (OR = 1.99, 95% CI 1.01–3.91). Dashes mark unavailable output values of logistic regression models for SNPs not in HWE.

**Table 3:** Logistic regression models demonstrating the combined effects of the examined SNPs on COVID-19 susceptibility and neurological symptoms.

Predictor	Reference Group	Analyzed Group	SARS-CoV-2 Infection			Accompanying Neurological Symptoms			Long-Term Neurological Symptoms		
			β	S.E.	*p*-Value	β	S.E.	*p*-Value	β	S.E.	*p*-Value
Sex	Men	Women	−0.58	0.35	0.099	-	-	-	-	-	-
*FURIN* rs1894401	*AA*	*GG+GA*	0.85	0.29	0.0038	-	-	-	0.58	0.34	0.088
*SLC6A20* rs2531743	*GG*	*AA+AG*	0.66	0.29	0.026	-	-	-	0.75	0.34	0.031
*NR1H2* rs1405655	*TT*	*CC*	-	-	-	1.09	0.39	0.0059	-	-	-
*TMPRSS2* rs4290734	*AA*	*AG*	-	-	-	0.71	0.27	0.0099	-	-	-

Abbreviations: β–regression coefficient; S.E.–standard error. Dashes designate the predictors not-included in the regression model.

## Data Availability

The datasets used and/or analyzed in the current study are available from the corresponding author on reasonable request.

## Supplementary Materials

The following supporting information can be downloaded at: https://www.mdpi.com/article/10.3390/jpm13010123/s1, Table S1. A set of SNPs selected for the association analysis of COVID-19-related phenotypes in the present study. References [5,11,24] are cited in the supplementary materials.
