# Supplementary material for: Host Genetic Variants Linked to COVID-19 Neurological Complications and Susceptibility in Young Adults—A Preliminary Analysis
Source: J Pers Med. 2023 Jan 6;13(1):123. doi: 10.3390/jpm13010123 (PMC9860613; doi:10.3390/jpm13010123)
Supplement: Supplementary file 1 [file jpm-13-00123-s001.zip › jpm-2079395-supplementary.pdf]

**Table S1.** A set of SNPs selected for the association analysis of COVID-19-related phenotypes in the present study.

| SNP | Gene Name | Chr:position (GRCh37) | EA/NEA | Proxy GWAS SNP | HWE *p*-value | EA/NEA GWAS | Phenotype | Reference |
|---|---|---|---|---|---|---|---|---|
| rs1405655 | *NR1H2* | 19:50882619 | C/T | - | 0.80 | C/T | Hospitalized, AD | [1,2] |
| rs2109069 | *DPP9* | 19:4719443 | A/G | - | 0.46 | A/G | dementia | [1,3] |
| rs35705950 | *MUC5B* | 11:1241221 | T/G | - | 0.22 | T/G | Hospitalized, AD | [1,2] |
| rs505922 | *ABO* | 9:136149229 | C/T | - | 0.80 | T/C | dementia | [1] |
| rs10735079 | *OAS3* | 12:113380008 | G/A | - | 1 | G/A | dementia | [3] |
| rs2304256 | *TYK2* | 19:10475652 | A/C | rs11085727 | 1 | T/C | dementia | [3] |
| rs114067890 | *SMRR1* | 1:24999518 | G/A | rs111972040 | 1 | G/A | dementia | [3] |
| rs12329760 | *TMPRSS2* | 21:42852497 | T/C | - | 0.31 | T/C | dementia | [3] |
| rs112640945 | *CCHCR1* | 6:31117673 | T/C | rs143334143 | 0.11 | A/G | dementia | [3] |
| rs4290734 | *TMPRSS2* | 21:42853083 | G/A | rs17854725 | 0.10 | A/G | dementia | [3] |
| rs3131294 | *NOTCH4* | 6:32180146 | A/G | - | 0.27 | A/G | dementia | [3] |
| rs769449 | *APOE* | 19:45410002 | A/G | rs429358 | 0.53 | C/T | dementia | [3] |
| rs61735789 | *TMPRSS2* | 21:42852435 | A/G | - | 0.01 | A/G | dementia | [3] |
| rs117696554 | *TMPRSS2* | 21:42860532 | A/G | rs61735794 | <0.01 | T/C | dementia | [3] |
| rs1894401 | *FURIN* | 15:91429042 | G/A | rs6226 | 1 | G/C | dementia | [3] |
| rs4766664 | *OAS1* | 12:113362997 | T/G | rs6489867 | 0.75 | C/T | dementia | [3] |
| rs657152 | *ABO* | 9:136139265 | A/C | - | 0.04 | A/C | dementia | [3] |
| rs10911734 | *IVNS1ABP, SWT1* | 1:185422424 | T/C | rs6668622 | 0.49 | C/T | dementia | [3] |
| rs17078348 | *LZTFL1* | 3:45847241 | G/A | rs71325088 | 0.73 | C/T | dementia | [3] |
| rs7412 | *APOE* | 19:45412079 | T/C | - | 0.08 | T/C | dementia | [3] |
| rs2838046 | *TMPRSS2* | 21:42898366 | G/A | rs75603675 | <0.01 | A/C | dementia | [3] |
| rs9380142 | *HLA-G* | 6:29798794 | G/A | - | 0.19 | G/A | dementia | [3] |
| rs2531743 | *SLC6A20* | 3:45838300 | A/G | - | 0.06 | A/G | Hospitalized, AD | [2] |
| rs45524632 | *KEAP1* | 19:10596988 | A/C | - | 0.39 | A/C | critical Illness, AD | [2] |

| | | | | | | | | |
|---|---|---|---|---|---|---|---|---|
| rs635634 | *ABO* | 9:136155000 | T/C | - | <0.01 | T/C | critical Illness, AD | [2] |

EA/NEA – effect (minor) allele/non-effect (major) allele; AD – Alzheimer’s disease.

References:


1. COVID-19 Host Genetics Initiative. Mapping the Human Genetic Architecture of COVID-19. *Nature* **2021**, *600*, 472–477. http://doi:10.1038/s41586-021-03767-x.

2. Baranova, A.; Cao, H.; Zhang, F. Causal Effect of COVID-19 on Alzheimer’s Disease: A Mendelian Randomization Study. *J. Med. Virol.* **2022**, http://doi:10.1002/jmv.28107.

3. de Rojas, I.; Hernández, I.; Montrreal, L.; Quintela, I.; Calero, M.; Royo, J.L.; Huerto Vilas, R.; González-Pérez, A.; Franco-Macías, E.; Macías, J.; et al. Genomic Characterization of Host Factors Related to SARS-CoV-2 Infection in People with Dementia and Control Populations: The GR@ACE/DEGESCO Study. *J. Pers. Med.* **2021**, *11*. http://doi:10.3390/jpm11121318.
